# Molecular Mechanisms of Dendritic Spine Development and Plasticity

**DOI:** 10.1155/2016/2078121

**Published:** 2016-04-03

**Authors:** Kwok-On Lai, Bryen A. Jordan, Xin-Ming Ma, Deepak P. Srivastava, Kimberly F. Tolias

**Affiliations:** ^1^School of Biomedical Sciences, State Key Laboratory of Brain and Cognitive Sciences, The University of Hong Kong, Hong Kong; ^2^Dominick P. Purpura Department of Neuroscience, Albert Einstein College of Medicine, Bronx, NY, USA; ^3^College of Life Science, Shaanxi Normal University, Xi'an, Shaanxi 710062, China; ^4^Department of Neuroscience, University of Connecticut Health Center, Farmington, CT 06030, USA; ^5^Department of Basic and Clinical Neuroscience, Institute of Psychiatry Psychology and Neuroscience, King's College London, London SE5 9RT, UK; ^6^Department of Neuroscience, Baylor College of Medicine, One Baylor Plaza, Houston, TX 77030, USA

Dendritic spines were first described by Santiago Ramón y Cajal more than one hundred years ago when he examined Golgi-stained cerebellar Purkinje cells of birds. Since then, considerable effort has been put towards understanding how these structures are formed and what their functions in the central nervous system are. It is now well established that dendritic spines represent specialized subcellular compartments on the postsynaptic neuron where the majority of excitatory synapses are located. Therefore, the density of dendritic spines is a rough indication of how much excitatory input a particular neuron receives. One notable feature of these structures is the large heterogeneity of their dimensions and shapes. They can exist as short stubby spines, long thin spines, and mushroom-shaped spines. Moreover, they are highly dynamic, such that ongoing spine growth, turnover, and morphological changes occur in both developing and adult brains. Although excitatory synapses can form and function outside of dendritic spines, their location on spine heads likely confers additional properties. For example, the presence of the spine neck is thought to create an isolated biochemical compartment on the spine head, where individual synapses of the postsynaptic neuron can function and be regulated independently of each other. Changes in the dimension and shape of individual spines also allow modulation of synaptic efficacy between specific neuronal partners and therefore contribute to synaptic plasticity and provide the cellular basis of learning and memory. Indeed, many molecular players that regulate dendritic spine morphogenesis also turn out to be essential for learning-related synaptic plasticity and memory formation. In this special issue, reviews and original research papers have been collected to address various questions on dendritic spine biology. These include the process of spine development, the functional differentiation of large and small spines, the relationships between spine changes and learning, the signaling pathways that control spine morphogenesis, and the link between spine abnormalities and brain disorders.

The formation of dendritic spines (spinogenesis) upon the initial axodendritic contact can be achieved through multiple ways as described by three different models (the Sotelo model, the Miller/Peters model, and the filopodial model). Much of the knowledge about spine formation is derived from studies on cortical, hippocampal, and cerebellar neurons. In this issue, R. Kanjhan et al. review the development of dendritic spines in motoneurons, which follows a different sequence than that described for pyramidal and Purkinje neurons in the brain. Using superoxide dismutase (SOD1) mutant mice as example, the article further discusses increases in spine density and hyperexcitability as potential causes of motoneuron degenerative disorders.

Dendritic spines come in diverse sizes and morphologies. It is generally believed that spines with larger spine heads have greater synaptic strength than smaller spines. However, whether spines with different sizes serve distinct functions is not clear. J. J. W. Paulin et al. investigate the behavior of large and small spines within the same dendritic segments in response to tetraethylammonium chloride (TEA), which induces long-term potentiation in CA1 hippocampal neurons. They find that TEA induces opposite changes on small and large spines. The authors suggest that the immediate shrinkage of large spines is crucial for homeostatic protection whereas the subsequent enlargement of small spines is involved in synaptic strengthening.

Spine enlargement and stabilization require the synthesis of new proteins. Specific subsets of mRNAs are actively transported by specific RNA-binding proteins from the cell body to neuronal dendrites, where local protein synthesis is triggered by synaptic activity and contributes to enhanced synaptic strength in a synapse-specific manner. By pairing electric shock to whisker stimulation, M. Jasinska et al. examine the morphological changes of spines in the barrel cortex triggered by associative learning. They observe that fear conditioning increases the number of spines that contain a spine apparatus, a smooth ER-related membrane structure that may be involved in local protein synthesis and calcium buffering, as well as the number of polyribosomes in ER-free, single-synapse spines. These results support the notion that learning can increase the capacity of local protein synthesis near relevant synapses. M. E. Klein et al. review the recent advances in understanding of the role of RNA-binding proteins in neuronal targeting of mRNAs and synaptic plasticity. The authors also discuss the link between unbalanced local protein synthesis and degradation and various neurodevelopmental disorders. The article raises many open questions on this exciting and challenging field that warrant further investigation.

Although the molecular mechanisms underlying dendritic spine morphogenesis have been extensively studied in the past decade, information on this topic is still limited. Emerging studies have revealed many novel signaling mechanisms in the regulation of dendritic spine morphogenesis. G proteins are highly expressed in the brain, and specific G*α* subunits are present in the postsynaptic density. V. T. Ramírez et al. demonstrate that activation of pertussis toxin-sensitive G proteins by the peptide mastoparan promotes the formation of dendritic spines and PSD-95 clusters in hippocampal neurons through a CaMKII-dependent mechanism. Numerous G-protein-coupled receptors (GPCRs) are encoded by the human genome, and one subfamily is the brain-specific angiogenesis inhibitor (BAI) subfamily of adhesion-GPCRs, which contains multiple domains in the N-terminal region that bind to other cells or the extracellular matrix. J. G. Duman et al. review recent findings that support an important role of this family of GPCRs in regulating spine morphogenesis, synaptic plasticity, and memory formation. BAI1 mediates its action through a complex of Par3 and Tiam1. Par3 belongs to the partitioning-defective proteins (Par family) that are well-known cell polarity determinants. The review by H. Zhang summarizes the emerging functions of Par proteins as well as other cell polarity complexes (the septin GTPases and Planar cell polarity proteins such as Frizzled, Dishevelled, and Van Gogh) in dendritic spine development and plasticity. Tiam1, on the other hand, is a guanine nucleotide exchange factor (GEF) for the small GTPase Rac1. The activity of GTPases is determined by GEFs, which catalyze the exchange of GDP to GTP, and GAPs, which terminate GTPase activity by hydrolyzing GTP. K. M. Woolfrey and D. P. Srivastava review how the Rho and Ras families of small GTPases and their upstream regulators act as signaling hubs to regulate dendritic spine morphogenesis in response to diverse extracellular stimuli.

Dendritic spines are supported by the actin cytoskeleton, and the function of many small GTPases on spine morphogenesis is mediated by changes in actin dynamics. Using Fluorescence Recovery After Photobleaching (FRAP), N. Domínguez-Iturza et al. examine the actin dynamics of individual spines in both cultured neurons and organotypic slices. The authors find that individual spines display specific actin dynamics independent of their positions and therefore consistent with the notion that the structure of each spine can be independently regulated. However, actin mobility within spine heads depends on contacts with astrocytes as well as spine size. Actin binds to many different proteins. One of them is the collapsing response mediator protein 2 (CRMP2), which also binds to tubulin and regulates microtubule assembly. CRMP2 can be phosphorylated by the proline-directed serine/threonine kinase Cdk5, an important kinase that regulates synapse development and function. X. Jin et al. investigate the significance of this phosphorylation event by generating CRMP2 knock-in mice, in which the Cdk5 phosphorylation site Ser-522 is substituted by Ala and therefore becomes phosphorylation-deficient. The authors observe spine loss in CA1 hippocampal neurons of the knock-in mice and suggest that it will be important to further delineate whether the CRMP2 phosphorylation mediates its effect on actin or tubulin.

Dendritic spine abnormalities have been frequently associated with various neurodevelopmental disorders such as Autism Spectrum Disorder (ASD) and intellectual disabilities. Using the heparin sulfate proteoglycan syndecan-2 and the neuron-specific F-actin regulator cortactin-binding protein 2 (CTTNBP2) as examples, H.-T. Hu et al. review how defects in neuron-specific signal transduction pathways underlying dendritic spine morphogenesis and synapse formation might contribute to the pathogenesis of these disorders. Altered spine density and morphology are also linked to psychiatric disorders such as depression. H. Qiao et al. review the three animal models of chronic stress; two of them have been widely used as an animal model of depression for recapitulating depression-like behaviors in rodents and studying the mechanisms underlying depression. Chronic stress generally causes dendritic atrophy and spine loss in the hippocampus and prefrontal cortex and yet an increase in spine density in the amygdala and nucleus accumbens. These alterations of dendritic spines are often accompanied by depression-like behaviors. The putative mechanisms that underlie the stress-induced changes in synapse structure are also discussed.

By highlighting the molecular basis of dendritic spine morphogenesis and plasticity, we hope this special issue will provide a better understanding of learning and memory, as well as other higher-order cognitive functions of the adult brain. Insights into the mechanisms behind altered spine morphogenesis in various neurodevelopmental and psychiatric disorders may further lead to the design of potential therapeutic strategies.

*Kwok-On Lai* *Kwok-On Lai* *Bryen A. Jordan* *Bryen A. Jordan* *Xin-Ming Ma* *Xin-Ming Ma* *Deepak P. Srivastava* *Deepak P. Srivastava* *Kimberly F. Tolias* *Kimberly F. Tolias*

